# Evaluation of Semicircular Canal Function Using Video Head Impulse Test in Patients With Peripheral Vestibular Disorders Without Nystagmus

**DOI:** 10.7759/cureus.62786

**Published:** 2024-06-20

**Authors:** Keishi Fujiwara, Shinya Morita, Kimiko Hoshino, Atsushi Fukuda, Hideaki Takeda, Yuji Nakamaru, Akihiro Homma

**Affiliations:** 1 Department of Otolaryngology - Head and Neck Surgery, Faculty of Medicine and Graduate School of Medicine, Hokkaido University, Sapporo, JPN

**Keywords:** vestibular compensation, caloric testing, nystagmus, catch-up saccade, video head impulse test

## Abstract

Objectives

This study aims to evaluate semicircular canal function using video head impulse test (vHIT) in patients with peripheral vestibular disorders without nystagmus.

Methods

Patients who underwent vHIT were enrolled in this study, and the proportion of abnormal findings on vHIT in patients without nystagmus was investigated. In addition, the results of vestibular testing were investigated in cases in which both vHIT and caloric testing were performed in patients without nystagmus.

Results

Forty-six patients (23.4%) of 197 patients who had no abnormal findings on the nystagmus tests, including the gaze nystagmus test, positional nystagmus test, and positioning nystagmus test, showed dysfunction in at least one semicircular canal on vHIT. The most frequent diagnosis was vestibular schwannoma (14/46, 30.4%), and cases with bilateral vestibular dysfunction were also included (12/46, 26.1%). A disorganized pattern of catch-up saccade was observed more frequently in patients with subjective symptoms of dizziness/vertigo compared to those without subjective symptoms. Although the sensitivity of vHIT was low compared to caloric testing, vHIT could detect isolated vertical canal dysfunction not detected by caloric testing.

Conclusions

vHIT is considered to be a useful test for patients without nystagmus, as vHIT could detect abnormalities in approximately one-quarter of patients without nystagmus. vHIT is considered to be one of the first tests to be performed following nystagmus testing, including the gaze nystagmus test, the positional nystagmus test, and the positioning nystagmus test. On the other hand, there are some cases in which vHIT shows no abnormality while caloric testing shows canal paresis. It is necessary to perform vHIT, bearing in mind that there are abnormalities that cannot be detected by vHIT alone.

## Introduction

In recent years, there have been significant advances in tests of vestibular function, including video head impulse test (vHIT) [1], vestibular-evoked myogenic potentials [2,3], and endolymphatic hydrops detection by MRI [4]. As a result of these advances, the proportion of cases diagnosed as vertigo of unknown origin is decreasing [5]. On the other hand, detailed vestibular examinations, including caloric testing, are time-consuming; therefore, it is not possible to perform these tests on all cases complaining of vertigo/dizziness, and cases for detailed examination should be appropriately selected.

Nystagmus testing is one of the first tests performed in the examination of patients with vertigo [6] and consists of gaze nystagmus testing, positional nystagmus testing, positioning nystagmus testing, head-shaking-induced nystagmus testing, hyperventilation-induced nystagmus testing, and vibration-induced nystagmus testing. If nystagmus is detected using these tests, the patient is presumed to have an abnormality somewhere in the vestibular pathway. Nystagmus may be of central or peripheral origin [7]. However, if no nystagmus is detected, the patient might be diagnosed as not having any peripheral vestibular disorder. Nevertheless, some patients with peripheral vestibular disorders, such as those with bilateral vestibulopathy, may not have nystagmus. Compared to caloric testing, which has been the gold standard test for patients with vertigo/dizziness, vHIT is less time-consuming and less invasive [8]. Moreover, vHIT can evaluate vertical canal function [9] and is considered to be a screening test for peripheral vestibular disorders [10]. In this retrospective study, we evaluated vHIT findings in patients without nystagmus, and the usefulness of vHIT as a screening test was investigated.

## Materials and methods

Patients

We retrospectively reviewed the medical records of patients who underwent vHIT in the Department of Otolaryngology at Hokkaido University Hospital between March 2017 and October 2023. The results of vHIT and nystagmus tests, including gaze, spontaneous, positional, and positioning nystagmus tests, were confirmed, and the proportion of abnormal findings on vHIT in patients without nystagmus was investigated. In addition, the results of vestibular testing were investigated in cases in which both vHIT and caloric testing were performed in patients without nystagmus.

Patients without subjective vestibular symptoms at the time of examination, for example, those with fluctuating vertigo, such as Meniere's disease, or with no subjective symptoms of vestibular schwannoma, were included. The patients in whom vHIT of the vertical semicircular canals was not performed were also included. Patients in whom a sufficient number of vHIT impulses (≥20) were not performed in any of the three planes (the horizontal, left anterior-right posterior (LARP), and right anterior-left posterior (RALP)) due to factors such as poor pupil recognition or cervical spine disease were excluded.

This research adhered to the tenets of the Declaration of Helsinki and was approved by our Institutional Review Board of Hokkaido University Hospital (approval number: 023-0163). Informed consent was obtained from all individual participants included in the study.

Nystagmus tests

Gaze nystagmus was tested without a charge-coupled device (CCD) camera, and spontaneous, positional, and positioning nystagmus were tested in the dark using a CCD camera. A head-shaking test was also performed.

vHIT

**Figure 1:**
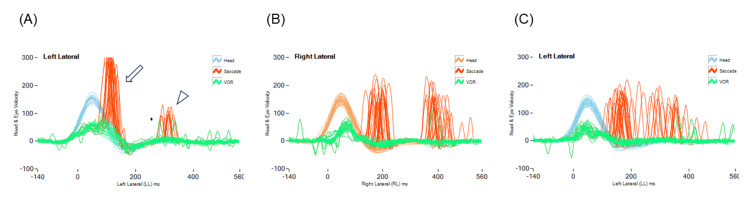
Representative results for CUS pattern (A) Covert and overt CUS. CUS was classified as covert if the onset occurred before the end of the head impulse (arrow) and as overt if it occurred afterward (arrowhead). (B) Organized CUS pattern. CUS occurred at the same time interval, showing small variability in the latency. (C) Disorganized CUS pattern. CUS occurred randomly over the testing time. CUS: catch-up saccade

Our vHIT methodology has been described previously [11]. Briefly, the semicircular canal function was evaluated using an ICS impulse 3D video head impulse system (GN Otometrics, Taastrup, Denmark). The head of the subject was quickly turned (amplitude of 10-20º) with unpredictable timing and direction corresponding to the horizontal, LARP, and RALP planes, and the vestibulo-ocular reflex (VOR) gains in the anterior semicircular canal (ASC), horizontal semicircular canal (HSC), and posterior semicircular canal (PSC) were evaluated. The VOR gain, i.e., the ratio of the area under the curves for head and eye velocity, was calculated from the average of at least 20 head impulses. Trials with blinks and outliers were detected automatically or manually and excluded from the recording. A normal gain was defined as ≥0.80 for the horizontal canal and ≥0.70 for the vertical canals [12]. The catch-up saccade (CUS) was defined as abnormal in this study if the velocity of the CUS exceeded 90 deg/sec based on our previous study [11]. CUS was classified as covert if the onset occurred before the end of the head impulse and as overt if it occurred afterward (Figure 1A) [13]. As a further classification, CUS was classified as organized or disorganized [14]. Organized CUS occurred at the same time interval in at least 80% of impulses performed, showing small variability in the latency, and disorganized CUS occurred randomly over the time period registered (Figure 1B-1C) [14]. In this study, the judgment of semicircular canal dysfunction was made when a reduction in VOR gain and the presence of CUS were observed. No severe adverse events were associated with vHIT. All examinations were performed by the same operator.

Caloric testing

Monothermal caloric testing using cold water (20°C) was performed [15,16]. Eye movements were recorded by video-oculography (Meditester VOG; Panasonic, Osaka, Japan), and the maximum slow-phase eye velocities (MSPV) were calculated. An MSPV <10°/s bilaterally or CP% > 20 was regarded as indicating a bilateral or unilateral weakness in responses, respectively.

Statistical analysis

Statistical analyses were performed using GraphPad Prism software (version 6.0; GraphPad Software Inc., La Jolla, CA). Statistical differences were analyzed using the chi-square test, the McNemar test, the Mann-Whitney U test, and the Fisher’s exact test, with a p-value of less than 0.05 considered statistically significant.

## Results

Subject profiles

**Figure 2:**
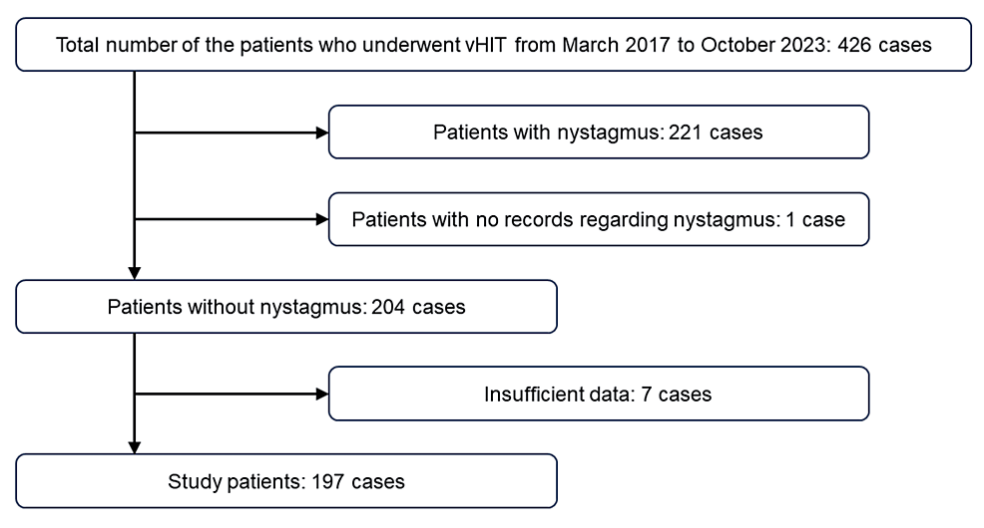
Study flowchart Of 426 patients, 222 were excluded because of findings of nystagmus. Of the 204 cases without nystagmus, seven were excluded due to insufficient vHIT data. vHIT: video head impulse test

As shown in the study flowchart (Figure 2), 426 consecutive patients underwent vHIT during the study period. Of these, some kind of abnormal finding on the nystagmus tests was detected in 221 patients, with no record of the nystagmus tests available for one patient. Therefore, 204 patients had no abnormal findings on the nystagmus tests. Seven patients were excluded due to an insufficient number of vHIT impulses, so the results of vHIT were investigated in 197 patients without nystagmus. Detailed subject profiles are shown in Table 1. Seventy-six patients were male, with a median age of 52 years (range: 8-88 years). Subjective symptoms of dizziness/vertigo at the time of examination were reported in 121 patients, and 76 patients reported no symptoms of dizziness/vertigo. Twenty-three patients underwent horizontal vHIT only, and either LARP or RARP was lacking in nine cases. All three planes (horizontal, LARP, and RALP) were examined in 165 cases.

**Table 1:** Detailed subject profiles The data is represented as n (%). vHIT: video head impulse test, LARP: left anterior-right posterior, RALP: right anterior-left posterior

Subject profiles	
Age (y)	
Range	8-88
Median	52
Gender (n, %)	
Male	76 (38.6%)
Female	121 (61.4%)
Subjective vestibular symptoms (n, %)	
Yes	121 (61.4%)
No	76 (38.6%)
vHIT data	
Horizontal only	23 (11.7%)
Lack of LARP or RALP	9 (4.6%)
Full	165 (83.8%)

vHIT findings in patients without nystagmus

Of the 197 patients without nystagmus, 46 (23.4%) showed dysfunction in at least one semicircular canal on vHIT based on the criteria described above. The most frequent diagnosis was vestibular schwannoma in 14 (30.4%), followed by cholesteatoma with labyrinthine fistula in five, otitis media with antineutrophilic cytoplasmic antibody (ANCA)-associated vasculitis in four, neurofibromatosis type 2 in three, and meningeal carcinomatosis in two patients. Diagnoses are listed in Table 2.

**Table 2:** Diagnoses of the patients without nystagmus in whom semicircular canal dysfunction was observed The data is represented as n (%). OMAAV: otitis media with antineutrophilic cytoplasmic antibody-associated vasculitis, CPA: cerebellopontine angle

Diagnoses of the patients, no. (%)	
Vestibular schwannoma	14 (30.4)
Cholesteatoma with labyrinthine fistula	5 (10.9)
OMAAV	4 (8.7)
Neurofibromatosis type 2	3 (6.5)
Meniere’s disease	2 (4.3)
Peripheral vestibular disease	2 (4.3)
Meningeal carcinomatosis	2 (4.3)
Stenosis of the internal auditory canal	1 (2.2)
Delayed endolymphatic hydrops	1 (2.2)
Vestibular paroxysmia	1 (2.2)
Vestibular migraine	1 (2.2)
Intralabyrinthine schwannoma	1 (2.2)
Meningioma in CPA	1 (2.2)
Relapsing polychondritis	1 (2.2)
Eosinophilic otitis media	1 (2.2)
Bell’s palsy	1 (2.2)
Gentamicin-induced vestibulotoxicity	1 (2.2)
Aortitis syndrome	1 (2.2)
Meningitis	1 (2.2)
Vertigo/dizziness of unknown origin	2 (4.3)

Abnormal findings on vHIT were detected bilaterally in 12 cases and unilaterally in 34 cases. Abnormal findings in the vertical canals were only detected in 20 cases, and abnormal findings in the horizontal canal were only detected in 10 cases. Sixteen cases showed abnormal findings in both vertical and horizontal canals. There was no significant difference in age between the two groups, those with and without abnormal findings on vHIT (p=0.88, Mann-Whiteny U test). When the detection rate of abnormal findings on vHIT was examined in the presence or absence of subjective symptoms of dizziness/vertigo, 28 out of 121 patients with symptoms had abnormal findings on vHIT, and 18 out of 76 patients without symptoms had abnormal findings on vHIT, with no significant difference observed between the two groups (p=0.93, chi-square test).

Relationship between vHIT findings and subjective symptoms

**Figure 3:**
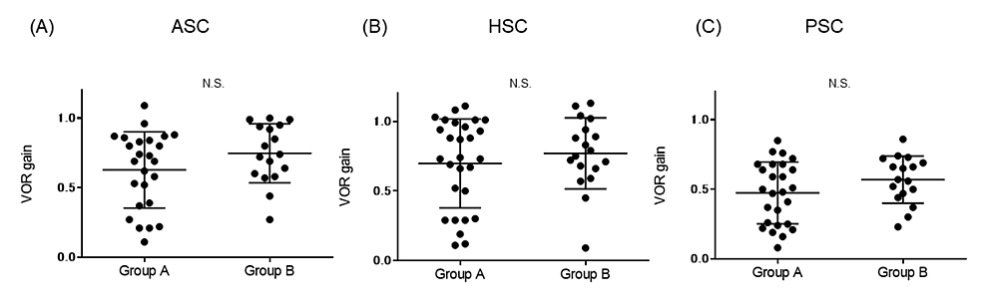
Comparison of VOR gain in the affected ear between Group A and B VOR gain in ASC (A), HSC (B), and PSC (C) of each group are shown. There were no significant differences for any of the three canals. VOR: vestibulo-ocular reflex, ASC: anterior semicircular canal, HSC: horizontal semicircular canal, PSC: posterior semicircular canal

Forty-six patients with abnormal findings on vHIT were divided into two groups based on the presence or absence of subjective symptoms. Detailed vHIT findings are shown in Table 3. Dysfunction was detected in 60 semicircular canals in the 28 cases with subjective symptoms (Group A), and dysfunction was detected in 26 semicircular canals in the 18 cases without subjective symptoms (Group B). The ratio of bilateral dysfunction did not differ significantly between the two groups (p=0.32, Fisher’s exact test). When comparing the VOR gain in the affected ear (or the worse ear in cases with bilateral dysfunction), there were no significant differences between the VOR gain of the ASC, HSC, and PSC between the two groups (p=0.17, p=0.60, and p=0.17, respectively, Mann-Whiteny U test) (Figure 3A-3C). In the patients with subjective symptoms, covert CUS was detected in two semicircular canals, overt CUS was detected in 30 semicircular canals, and both covert and overt CUS were detected in 28 semicircular canals, while an organized pattern was detected in 23 semicircular canals and a disorganized pattern was detected in 37 semicircular canals. In the patients without subjective symptoms, covert CUS was detected in two semicircular canals, overt CUS was detected in 13 semicircular canals, and both covert and overt CUS were detected in 11 semicircular canals, while an organized pattern was detected in 20 semicircular canals and a disorganized pattern was detected in six semicircular canals. Although there was no significant difference in the ratio of covert, overt, and both covert and overt CUS between the two groups (p=0.66, chi-square test), there was a significant difference in the ratio of organized and disorganized patterns between the two groups (p=0.0019, Fisher’s exact test).

**Table 3:** Detailed vHIT findings Dysfunction was detected in 60 semicircular canals in 28 cases with subjective symptoms (Group A) and dysfunction was detected in 26 semicircular canals in 18 cases without subjective symptoms (Group B). The CUS pattern of each group is shown. The data is represented as n (%). Group A: patients with subjective symptoms, Group B: patients without subjective symptoms SCC: semicircular canal, CUS: catch-up saccade, Bil: bilateral, Uni: unilateral, C: covert, O: overt, Org: organized, Disorg: disorganized

	Cases	Affected side			Affected SCC	CUS pattern			CUS pattern		
Group A	28	Bil	9	(32.1%)	60	C	2	(3.3%)	Org	23	(38.3%)
		Uni	19	(67.9%)		O	30	(50.0%)	Disorg	37	(61.7%)
						C + O	28	(46.7%)			
Group B	18	Bil	3	(16.7%)	26	C	2	(7.6%)	Org	20	(76.9%)
		Uni	15	(83.3%)		O	13	(50.0%)	Disorg	6	(23.1%)
						C + O	11	(42.3%)			

Comparison of the results of vHIT and caloric testing

Of 196 patients without nystagmus, caloric testing was performed in 78 patients. The results of vHIT and caloric testing were compared in these 78 patients. Forty patients (51.3%) were defined as having a unilateral or bilateral weakness in responses to caloric testing. On the other hand, semicircular canal dysfunction was detected by vHIT in 16 (20.5%) of the 78 patients. Although consistency between vHIT and caloric test results was observed in 46 cases, discrepancies between the results of the two tests were observed in 32 cases, and there was statistical significance between vHIT and caloric testing (p<0.0001, McNemar test) (Table 4). The patterns of discrepancy were vHIT normal (caloric testing abnormal in 28 cases) and vHIT abnormal (caloric testing normal in four cases). Of the four cases in which vHIT was abnormal and caloric testing was normal, normal findings were observed in the horizontal canal, whereas dysfunction in the posterior canal was detected on vHIT in three cases. These cases indicated that isolated PSC dysfunction, which was not detected by caloric testing, could only be detected by vHIT. vHIT has a sensitivity of 0.30, a specificity of 0.89, a positive predictive value of 0.75, and a negative predictive value of 0.55 against caloric testing as the gold standard.

**Table 4:** Comparison of the results of vHIT and caloric testing Although consistency in vHIT and caloric test results was observed in 46 cases, discrepancies between the results of the two tests were observed in 32 cases. There was a statistical significance between vHIT and caloric testing (p<0.0001, McNemar test). The data is represented as n (%). vHIT: video head impulse test

vHIT	Caloric testing		
	Abnormal	Normal	Total
Abnormal	12	4	16
Normal	28	34	62
Total	40	38	78

## Discussion

In the present study, of the patients without nystagmus, 23.4% had abnormal findings on vHIT. The most common diseases included vestibular schwannoma and cholesteatoma with labyrinthine fistula. In routine clinical practice, nystagmus tests could be the quickest method of screening for vestibular dysfunction [17]; however, a number of patients with vertigo/dizziness do not have nystagmus. Whether or not there are abnormalities in the vestibular system in these patients needs to be investigated by a detailed examination. The present study demonstrates the usefulness of vHIT in detecting semicircular canal dysfunction in patients without nystagmus.

Although vHIT is considered to be less time-consuming and less invasive than caloric testing, vHIT has low sensitivity compared to caloric testing [8]. Of the 78 patients who underwent both vHIT and caloric testing, 28 patients (35.9%) had an abnormal result on caloric testing and a normal result on vHIT, and the sensitivity of vHIT against caloric testing was quite low at 0.30. It has been reported that discrepancies between the results of caloric testing and vHIT were observed in patients with endolymphatic hydrops [18]. Therefore, even if the results of vHIT are normal, this doesn't mean that the results of caloric testing will also be normal. West et al. reported that vHIT is sensitive to vestibular schwannoma but less so than caloric testing [19]. Therefore, vHIT does not afford a substitute for caloric testing, and these tests should be regarded as complementary in assessing patients with dizziness [20], with the results of the present study in patients without nystagmus also supporting this. It has been suggested that additional caloric testing is indicated in cases of vHIT normality, while an abnormal vHIT result is strongly related to an abnormal caloric test result [21]. From these results, it can be concluded that vHIT is clinically useful as a first test for determining vestibular hypofunction in patients with dizziness [21]. Although this study includes only a small number of cases, vHIT could detect vertical semicircular canal dysfunction in patients with normal caloric test results. One of the advantages of vHIT over caloric testing is that it can evaluate the function of the vertical semicircular canals, whereas caloric testing only evaluates the function of the HSC [9,11].

The time course of dynamic vestibular compensation in patients with unilateral peripheral vestibular loss was investigated using head-shaking-induced nystagmus [22]. Spontaneous nystagmus disappeared, and head-shaking-induced nystagmus decreased significantly from two years after onset due to vestibular compensation, whereas vHIT could detect a vestibular deficit after two years [22]. The patients without nystagmus but with semicircular canal dysfunction on vHIT are thought to include cases in whom nystagmus disappeared due to vestibular compensation but semicircular canal dysfunction was still present. Nystagmus testing alone is insufficient for the detection of such cases, and vHIT could be useful. If semicircular canal dysfunction was detected by vHIT in patients without nystagmus, vestibular rehabilitation could improve the subjective symptoms in these patients [23]. In this study, the most common diagnosis was vestibular schwannoma. Patients with vestibular schwannoma often have no nystagmus due to vestibular compensation. However, the function of the semicircular canal or vestibular nerve remains impaired, and abnormal findings are detected on vHIT. This was thought to be a reason for the high proportion of cases with vestibular schwannoma.

In this study, 12 of 46 cases (26.1%) showed bilateral vestibular dysfunction on vHIT. Bilateral vestibular dysfunction has a variety of etiologies, including idiopathic, bilateral Meniere’s disease, gentamicin-induced vestibulotoxicity, or meningitis [24]. Although rare, neurofibromatosis type 2, meningeal carcinomatosis, and autoimmune diseases, which were observed in the patients without nystagmus in this study, were also reported as the causes of bilateral vestibulopathy [24]. It has been reported that 70% of patients with bilateral vestibulopathy show no nystagmus; however, semicircular canal dysfunction can be detected by vHIT in all cases [25]. In the present study, cases of bilateral vestibular disorders were included among the cases with balance disorders without nystagmus, suggesting the usefulness of vHIT in detecting bilateral vestibular disorders.

In the present study, the 46 patients in whom semicircular dysfunction was detected by vHIT were divided into two groups: those with or without subjective symptoms of dizziness or vertigo. VOR gain, unilateral or bilateral dysfunction, and single or multiple dysfunctions showed no significant correlations with subjective symptoms. An organized CUS pattern was detected at a significantly greater rate in patients without subjective symptoms compared with those with symptoms. There are some reports on the relationship between CUS and vestibular compensation, and it has been reported that a disorganized CUS pattern in vHIT was observed together with higher DHI scores in patients with vestibular deafferentation [26], and patients with a disorganized CUS pattern showed a higher level of vestibular disability and handicap after vestibular schwannoma surgery [14]. Consistent with these previous reports, a disorganized CUS pattern was observed significantly more frequently in the group with subjective symptoms of dizziness in the present study. It has been speculated that vestibular compensation may not be adequate in patients with a disorganized CUS pattern, as these patients could not organize the eye response during unpredicted impulsive head movements [14]. If an organized CUS pattern represents better compensation than a disorganized pattern, patients without subjective symptoms can be considered to be well compensated, even though they have semicircular canal dysfunction.

The relationship between spontaneous nystagmus and vHIT findings has been evaluated in patients with chronic neurotologic conditions, with more than 90% of the patients reported as showing no abnormality on vHIT when they had no nystagmus [17]. In contrast to these results, the present study showed abnormal findings on vHIT in approximately one-quarter of patients without nystagmus. There are two possible reasons for this discrepancy. Firstly, the diagnoses of the patients included in the studies were different. In the previous report, Meniere’s disease was the most common disease, whereas only two cases of Meniere’s disease were included in the present study. Secondly, in the previous study, the HSC function was only investigated using vHIT. In the present study, only 43.4% of cases with vertical semicircular canal dysfunction were included among those with abnormalities on vHIT. The ability to evaluate the function of the vertical semicircular canals is one of the advantages of vHIT, and it is expected that including evaluation of the vertical semicircular canals improves the detection rate of abnormalities by vHIT. A number of cases of vHIT abnormalities without nystagmus have been reported in elderly patients [17]. As age-related VOR gain reductions have been reported [27], the effect of aging may be one of the reasons for the discrepancy between nystagmus and vHIT.

There are some limitations to this study. First, due to the retrospective nature of this study, the presence of bias based on the selection criteria for patients cannot be completely ruled out. Second, the time between the onset of symptoms and performing vHIT was not taken into account. Third, the number of cases in which caloric testing was performed was limited, which may lead to some bias. A prospective study should be conducted to investigate the usefulness of vHIT as a screening test in the future. Moreover, no patients with acute-phase vestibular disease were included in this study. Similar studies in hospitals with different target diseases (e.g., clinics) could yield interesting results.

## Conclusions

vHIT could detect abnormalities in approximately one-quarter of patients without nystagmus and is considered one of the first tests to be performed following nystagmus testing. On the other hand, there are some cases in which vHIT shows no abnormality but caloric testing shows canal paresis. It is necessary to perform vHIT, bearing in mind that there are abnormalities that cannot be detected by vHIT alone.
